# Association Between Stress Hyperglycemia Ratio and In-hospital Outcomes in Elderly Patients With Acute Myocardial Infarction

**DOI:** 10.3389/fcvm.2021.698725

**Published:** 2021-07-20

**Authors:** Guo Chen, Mingmin Li, Xiaodan Wen, Rui Wang, Yingling Zhou, Ling Xue, Xuyu He

**Affiliations:** ^1^Department of Cardiology, Guangdong Cardiovascular Institute, Guangdong Provincial Key Laboratory of Coronary Heart Disease Prevention, Guangdong Provincial People's Hospital, Guangdong Academy of Medical Sciences, Guangzhou, China; ^2^Department of Geriatrics, Guangdong Geriatrics Institute, Guangdong Provincial People's Hospital, Guangdong Academy of Medical Sciences, Guangzhou, China

**Keywords:** stress induced hyperglycemia, stress hyperglycemia ratio, acute myocardial infarction, elderly patients, in-hospital outcomes

## Abstract

**Backgrounds:** Emerging evidence suggests that stress hyperglycemia ratio (SHR), an index of relative stress hyperglycemia, is of great prognostic value in acute myocardial infarction (AMI), but current evidence is limited in elderly patients. In this study, we aimed to assess whether SHR is associated with in-hospital outcomes in elderly patients with AMI.

**Methods:** In this retrospective study, patients who were aged over 75 years and diagnosed with AMI were consecutively enrolled from 2015, January 1st to 2019, December 31th. Admission blood glucose and glycosylated hemoglobin (HbA1C) during the index hospitalization were used to calculate SHR. Restricted quadratic splines, receiver-operating curves, and logistic regression were performed to evaluate the association between SHR and in-hospital outcomes, including in-hospital all-cause death and in-hospital major adverse cardiac and cerebrovascular events (MACCEs) defined as a composite of all-cause death, cardiogenic shock, reinfarction, mechanical complications of MI, stroke, and major bleeding.

**Results:** A total of 341 subjects were included in this study. Higher SHR levels were observed in patients who had MACCEs (*n* = 69) or death (*n* = 44) during hospitalization. Compared with a SHR value below 1.25, a high SHR was independently associated with in-hospital MACCEs (odds ratio [OR]: 2.945, 95% confidence interval [CI]: 1.626–5.334, *P* < 0.001) and all-cause death (OR: 2.871 95% CI: 1.428–5.772, *P* = 0.003) in univariate and multivariate logisitic analysis. This relationship increased with SHR levels based on a non-linear dose-response curve. In contrast, admission glucose was only associated with clinical outcomes in univariate analysis. In subgroup analysis, high SHR was significantly predictive of worse in-hospital clinical outcomes in non-diabetic patients (MACCEs: 2.716 [1.281–5.762], *P* = 0.009; all-cause death: 2.394 [1.040–5.507], *P* = 0.040), but the association was not significant in diabetic patients.

**Conclusion:** SHR might serve as a simple and independent indicator of adverse in-hospital outcomes in elderly patients with AMI, especially in non-diabetic population.

## Introduction

Despite the marked progress in primary percutaneous coronary intervention (PCI) and medical management in the past decade, acute myocardial infarction (AMI) remains one of the leading causes of death worldwide (1). Numerous risk factors have been reported to be associated with adverse outcomes of AMI patients, among which chronic hyperglycemia has been well-documented among all age groups. Stress-induced hyperglycemia (SIH), an acute physiological response to stress, has also been identified as a strong predictor of mortality in critically ill patients (2–4). Interestingly, a higher mortality was observed in AMI patients with acute-onset of hyperglycemia than in those with chronic hyperglycemia, suggesting different mechanisms may mediate the extremely harmful effects of SIH (5, 6). However, the outcome-predicting significance of SIH in AMI patients varies among previous studies (7–9). The inconsistent results may be attributed to the fact that admission glucose concentrations were used as the index of SIH in these studies. Since the absolute admission glucose values could result from acute physiological stress, chronic high baseline glucose levels or both, it does not always accurately reflect the intensity of SIH (10).

Currently, nearly a third of patients admitted with AMI and two thirds dying from MI are over 75-year old (11), but elderly patients are under-represented in previous clinical studies, leading to limited evidence on the risk assessment and management strategy of this special subgroup. Moreover, comorbidities such as insulin resistance, frailty and malnutrition are common in elderly population (12, 13). While these factors also affect the baseline glycemic metabolism as well as acute response to stress (14, 15), the distinct spectra of glycemic status in older patients may influence the predictive value of SIH in AMI. Thus, a refined marker that takes into consideration of both chronic and acute glycemic status may better reflect the extent of SIH.

In recent years, stress hyperglycemia ratio (SHR) has been proposed as a better index of relative stress hyperglycemia, which is calculated from admission glucose adjusted for chronic glycemic status using glycosylated hemoglobin (HbA1c) (16). SHR is reported to be an independent predictor of poor prognosis in AMI patients undergoing PCI or discharge alive (17, 18). Until now, the prognostic value of SHR has not been clarified in elderly patients with AMI. Therefore, the aim of the present study was to investigate whether SHR is associated with in-hospital adverse outcomes and all-cause death in an elderly population with AMI.

## Materials and Methods

### Study Population

In this retrospective cohort study, we consecutively included subjects with an age ≥75 and who were diagnosed with AMI at the Cardiac Care Unit (CCU) of Guangdong Provincial People's Hospital from January 1st, 2014, to December 31st, 2019. The diagnosis of AMI was initially identified based on the principal discharge diagnosis, and the diagnosis of AMI was established if there were characteristic clinical symptoms of ischemia, electrocardiographic findings consistent with MI, and increased cardiac enzyme values that meet the Fourth Universal Definition of Myocardial Infarction (19). Patients who were lack of HbA1c values were excluded. The Institutional Review Board of Guangdong Provincial People's Hospital approved this retrospective study.

### Data Collection

Three experienced data inspectors collected information from medical records based on standardized definitions. Relevant data included demographic information, smoking status, medical history of hypertension, diabetes mellitus (DM), malignant tumors, prior MI or coronary revascularization, prior stroke, type of AMI (ST-segment elevation MI or non-ST-segment elevation), and cardiac arrest before admission. In addition, the Killip class, heart rate, systolic and diastolic blood pressure at admission, symptom-onset-to-balloon time (S2B), information about coronary revascularization, as well as left ventricular ejection fraction (LVEF) measured by echocardiography with Simpson's method, and multi-vessel disease were also recorded.

Laboratory biomarkers at admission including routine blood tests, N-terminal pro-brain natriuretic peptide (NT-proBNP), creatinine, lipid profiles, aminopherase were collected. All blood samples were run in real time for clinical purposes and were performed in the laboratory department (in accordance with the ISO 9000 Quality Management and Assurance Standards) at our medical center with standard examination methods.

### Outcomes

The primary observational outcome of this study was in-hospital major adverse cardiovascular and cerebrovascular events (MACCEs) defined as a composite of all-cause death, cardiogenic shock, reinfarction, mechanical complications of MI, ischemic stroke, and major bleeding. Cardiogenic shock was defined only for the patients who were initially not at shock status. Reinfarction was defined as a new AMI that occurred within 28 days of the index MI and met the criteria of the Fourth Universal Definition of Myocardial Infarction (19). Mechanical complications of AMI included papillary muscle rupture, ventricular free wall rupture, and ventricular septal rupture that occurred during the index hospitalization. Major bleeding was defined as the composite of clinically overt bleeding plus a drop in hemoglobin ≥5 g/dl, cardiac tamponade, any intracranial hemorrhage, and fatal bleeding (bleeding that directly results in death within 7 days). If a patient suffered several MACCE events during the index hospitalization, only one was counted in the calculation of MACCEs. The secondary outcome was defined as in-hospital all-cause death.

### Determination of Stress Hyperglycemia Ratio

The blood glucose on admission (ABG) was defined as the first available plasma glucose within 24 h of admission. HbA1c assays were performed during the index hospitalization using a blood analyzer (D-10, Bio-Rad Labrotories, CA, USA) equipped with a high-performance liquid chromatography system. SHR was defined as the admission glucose divided by the average glucose derived from HbA1c as follows: SHR = [(admission glucose (mg/dl))/(28.7 × HbA1c(%)−46.7)] (16). Patients who had a previous history of DM, or were taking anti-diabetic medications, or had a HbA1c over 6.5% were considered to have diabetes.

### Statistical Analysis

Data were summarized as mean ± standard deviation, number (%), and median [interquartile range (IQR)]. For comparison of clinical data between two groups, Mann–Whitney or unpaired *t*-tests were used for continuous data, and Pearson chi-square or Fisher's exact tests were used for categorical data as appropriate. To illustrate the relationship between SHR and the risk of in-hospital outcomes, we modeled SHR as restricted quadratic splines (RCS) with knots at the 5th, 35th, 65th, and 95th percentiles of its distribution to provide a smooth, yet flexible description of the dose–response relationship. A threshold for SHR of 1.25 is determined according to the combined consideration of RCS and receiver operating characteristic curves (ROC).

The associations between clinical variables and in-hospital outcomes were assessed by univariate logistic regression analysis. Clinical variables that were significant with a *P* < 0.05 in the univariate analysis, along with clinically important factors, were further assessed in the multivariate analysis with a forward stepwise regression method. A value of *P* < 0.05 (two-sided) was considered statistically significant in all tests. All data were analyzed with the SPSS. 26.0 (SPSS, Inc., Chicago, IL, USA) and R (version 3.4.3).

## Results

### Baseline Characteristics

From January 1st, 2014, to December 31st, 2019, a total of 2,404 patients were firstly diagnosed with AMI at the CCU. Among them, 356 patients were aged ≥75, and 15 patients lacking of HbA1c were excluded. Finally, 341 elderly patients with a diagnosis of AMI were included. The baseline clinical characteristics of these patients are shown in Table 1. The mean age of the study population was 80.67 ± 4.10 years, and 62.76% were male. Among them, 318 patients received PCI, while 23 patients received medical management only. During the index hospitalization, 69 patients had MACCEs (20.23%), and 44 patients died (12.90%). As shown in Table 1, SHR levels in patients with MACCEs or died were higher than those without MACCEs or mortality (*P* < 0.05).

**Table 1:** Baseline clinical characteristics of elderly patients with acute myocardial infarction.

**Variable**	**Overall**	**Non-MACCEs**	**MACCEs**	***P*-Value^**a**^**	**Survival**	**Death**	***P*-Value^**a**^**
	**(*n* = 341)**	**(*n* = 272)**	**(*n* = 69)**		**(*n* = 297)**	**(*n* = 44)**	
Age (years)	80.7 ± 4.1	80.5 ± 4.1	81.2 ± 4.1	0.199	80.6 ± 4.2	81.3 ± 3.7	0.179
Male (*n*, %)	214 (62.8)	177 (65.1)	37 (53.6)	0.079	191 (64.3)	23 (52.3)	0.123
Medical history (*n*, %)							
Current smoking	61 (17.9)	50 (18.4)	11 (15.9)	0.637	55 (18.5)	6 (13.6)	0.430
Diabetes	100 (29.3)	74 (27.2)	26 (37.7)	0.088	85 (28.6)	15 (34.1)	0.457
Hypertension	233 (68.3)	183 (67.3)	50 (72.5)	0.408	200 (67.3)	33 (75.0)	0.308
Prior MI, PCI, or CABG	73 (21.4)	54 (19.9)	19 (11.6)	0.165	61 (5.7)	12 (11.4)	0.309
Prior stroke	48 (14.1)	41 (15.1)	7 (10.1)	0.293	46 (15.5)	2 (4.5)	0.051
Clinical characteristics (*n*, %)							
STEMI	241 (70.7)	189 (69.5)	52 (75.4)	0.338	207 (69.7)	34 (77.3)	0.303
Killip class ≥ 2	153 (44.9)	109 (40.1)	44 (63.8)	<0.001^*^	123 (41.4)	31 (70.5)	<0.001^*^
Cardiac arrest before admission	13 (3.8)	7 (2.6)	6 (8.7)	0.029^b*^	9 (3.0)	4 (9.1)	0.072
Heart rate (per minute)	82.7 ± 17.3	81.1 ± 16.0	89.3 ± 20.5	0.001^*^	81.5 ± 16.5	90.6 ± 20.6	0.005^*^
SBP (mmHg)	124.6 ± 25.2	126.8 ± 24.0	115.9 ± 28.2	0.001^*^	125.8 ± 24.8	116.0 ± 26.9	0.009^*^
DBP (mmHg)	72.1 ± 14.8	72.7 ± 14.5	70.0 ± 16.2	0.064	72.5 ± 14.6	70.0 ± 16.1	0.113
Hospital stay (days)	8.0 (6.0–10.5)	8.0 (6.0–10.0)	9.0 (4.0–16.5)	0.145	8.0 (6.0–10.0)	6.5 (2.0–12.0)	0.065
Revascularization information (*n*, %)							
S2B within 12 h	170 (49.9)	144 (52.9)	26 (37.7)	0.024^*^	155 (52.2)	15 (34.1)	0.025^*^
Multi-vessel disease	246 (72.1)	197 (72.4)	49 (71.0)	0.815	216 (72.7)	30 (68.2)	0.530
PCI	318 (93.3)	259 (95.2)	59 (85.5)	0.012^*^	283 (95.3)	35 (79.5)	0.001^*^
Stent implantation	287 (84.2)	235 (86.4)	52 (75.4)	0.025^*^	256 (86.2)	31 (70.5)	0.008^*^
PTCA/thrombus aspiration only	31 (9.1)	27 (9.9)	4 (5.8)	0.287	24 (8.0)	7 (15.9)	0.097
Complete revascularization	215 (63.0)	181 (66.5)	34 (49.3)	0.008^*^	194 (65.3)	21 (47.7)	0.024^*^
Biochemical variables							
Glucose (mg/dl)	9.45 ± 3.99	9.15 ± 4.01	10.65 ± 3.73	<0.001^*^	9.28 ± 4.01	10.59 ± 3.74	0.010^*^
HbA1c (%)	6.48 ± 1.35	6.48 ± 1.42	6.47 ± 1.06	0.275	6.49 ± 1.41	6.41 ± 0.89	0.492
SHR	1.22 ± 0.39	1.19 ± 0.38	1.38 ± 0.41	<0.001^*^	1.20 ± 0.38	1.38 ± 0.41	0.003^*^
Hemoglobin (g/L)	124.23 ± 19.70	125.22 ± 19.03	120.36 ± 21.86	0.016^*^	125.04 ± 19.19	118.81 ± 22.32	0.040^*^
Creatinine (μmol/L)	119.26 ± 91.68	114.51 ± 94.15	137.95 ± 79.11	<0.001^*^	116.48 ± 93.16	137.97 ± 79.44	<0.001^*^
Peak CK	2042.74 ± 2011.02	1953.74 ± 1902.08	2394.84 ± 2377.42	0.350	1993.32 ± 1961.42	2380.63 ± 2319.72	0.435
Peak CK-MB	203.10 ± 212.62	195.53 ± 205.64	232.79 ± 237.42	0.365	196.38 ± 208.97	248.70 ± 233.53	0.134
Total bilirubin (mmol/L)	16.16 ± 7.42	15.79 ± 6.89	17.63 ± 9.13	0.188	15.84 ± 6.91	18.35 ± 10.08	0.210
ALT (U/L)	67.54 ±155.40	48.61 ± 55.35	142.17 ± 318.53	<0.001^*^	56.67 ± 127.67	140.94 ± 269.12	<0.001^*^
Total cholesterol (mmol/L)^c^	4.58 ± 1.09	4.62 ± 1.02	4.42 ± 1.31	0.240^c^	4.62 ± 1.06	4.31 ± 1.26	0.080^c^
Triglyceride (mmol/L)	1.33 ± 0.71	1.33 ± 0.71	1.36 ± 0.73	0.717	1.35 ± 0.74	1.18 ± 0.50	0.207
HDL-c (mmol/L)	1.06 ± 0.27	1.06 ± 0.25	1.07 ± 0.34	0.694	1.05 ± 0.25	1.13 ± 0.38	0.310
LDL-c (mmol/L)	2.93 ± 0.86	2.99 ± 0.83	2.72 ± 0.95	0.014^*^	2.97 ± 0.83	2.65 ± 0.96	0.023^*^
NT-proBNP (pg/ml)	3435 (1268, 8063,)	2696 (1128, 5830,)	7328 (3280, 1312,3)	<0.001^*^	2835(1154,7000)	6787 (3571, 1226,9)	<0.001^*^
LVEF (%)	47.72 ± 12.28	49.16 ± 11.70	42.04 ± 12.92	<0.001^*^	48.69 ± 11.93	41.18 ± 12.71	<0.001^*^

a *Mann-Whitney U-test or Pearson chi-square test*.

b *Fisher's exact tests*.

c *Unpaired t-test*.

* *P < 0.05*.

*MI, myocardial infarction; PCI, percutaneous coronary intervention; CABG, coronary artery bypass graft; STEMI, ST-segment elevation myocardial infarction; SHR, stress-induced hyperglycemia ratio; SBP, systolic blood pressure; DBP, diastolic blood pressure; S2B, symptoms to balloon time; PTCA, percutaneous transluminal coronary angioplasty; CK, creatine kinase; CK-MB, creatine kinase Isoenzyme-MB; ALT, alanine aminotransferase; HDL-c, high density lipoprotein cholesterol; LDL-c, low density lipoprotein cholesterol; NT-proBNP, N-terminal pro-brain natriuretic peptide; LVEF, left ventricular ejection fraction*.

The age, gender, smoking status, medical history, AMI types, and the incidence of multi-vessel disease were comparable among different patient groups. Compared to those without MACCEs, patients with MACCEs were less likely to receive PCI, stent implantation and to receive complete revascularization. Meanwhile, patients with MACCEs had longer S2B, higher incidence of cardiac arrest, higher Killip class, heart rate, serum creatinine, admission glucose, and NT-proBNP, while the systolic blood pressure (SBP), hemoglobin, and LVEF were lower. Likewise, the same differences in these clinical variables were observed between patients who died or not during the hospital stay.

### SHR and In-hospital Outcomes

**Figure 1:**
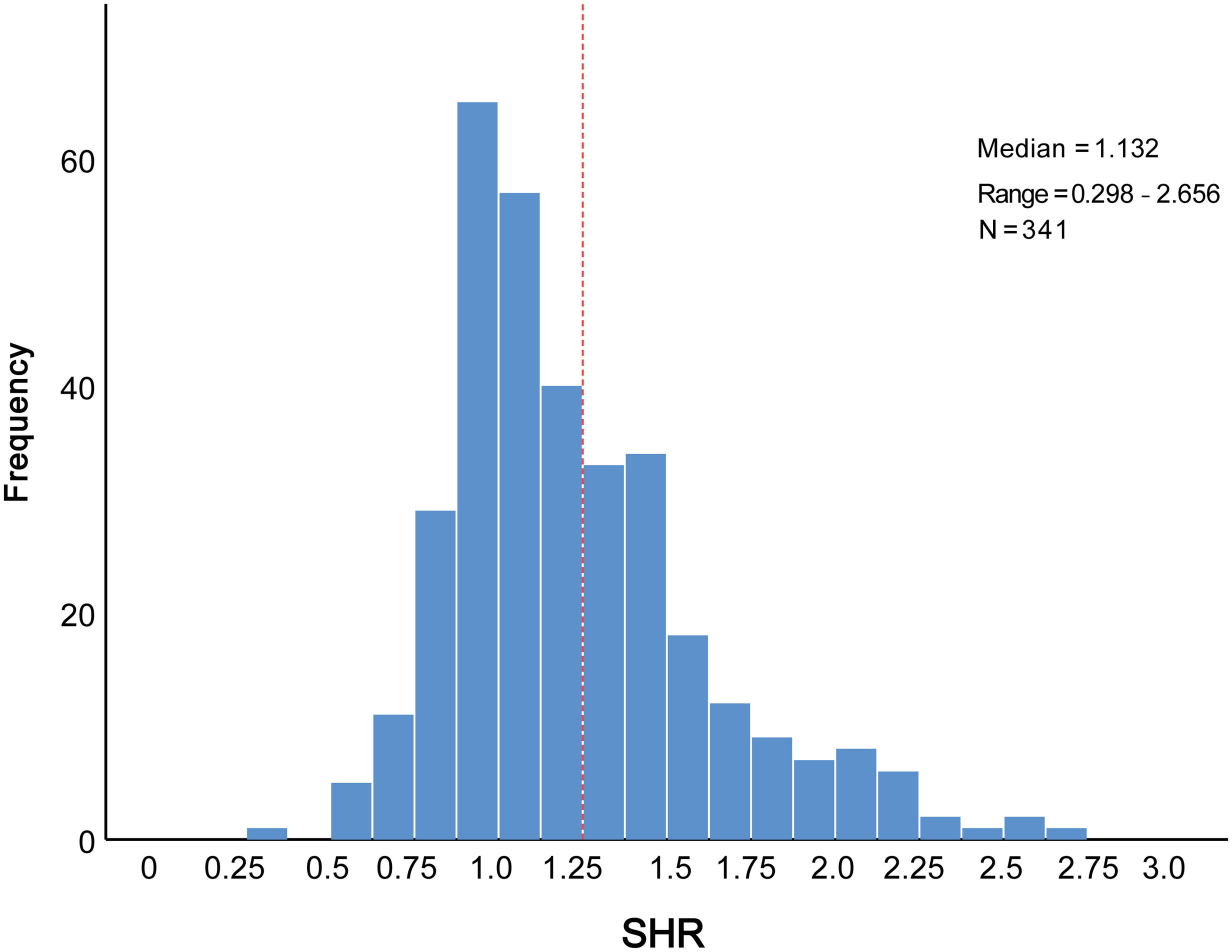
Distribution of SHR levels in the elderly patients with AMI. AMI, acute myocardial infarction; SHR, stress hyperglycemia ratio.

**Figure 2:**
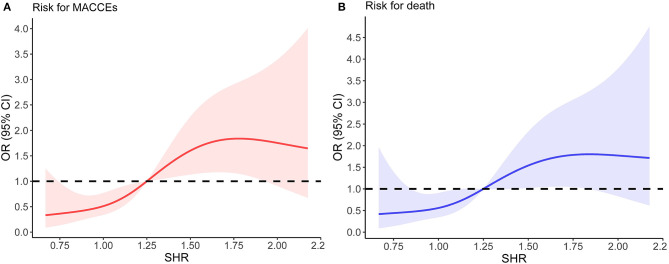
The relationship between SHR and the risk of in-hospital outcomes. **(A)** Non-linear dose–response relationship between SHR and in-hospital MACCEs. **(B)** Non-linear dose–response relationship between SHR and in-hospital mortality. In **(A,B)**, the x-axis is SHR level. The y-axis is the odds ratio, with the shaded area representing a 95% confidence interval. The threshold value of SHR (1.25) was set to 1.0 (referensce). OR, odds ratio; CI, confidence interval.

The distribution of SHR was displayed in Figure 1. To better illustrate the association between SHR distribution and clinical outcomes, we modeled SHR as RCS to provide a smooth, yet flexible description of their dose–response relationship. As displayed in Figure 2, SHR was associated with the risk of in-hospital MACCEs (Figure 2A, *P*-value for non-linear spline terms = 0.135) and death (Figure 2B, *P*-value for non-linear spline terms = 0.379) with a non-linear dose–response relationship. From receiver operating characteristic curves, a cut-off value of 1.20 was derived for in-hospital MACCEs (*P* < 0.001) and 1.32 for in-hospital death (*P* = 0.003). In order to provide a single SHR value for clinical use, a threshold of 1.25 was determined according to the combined consideration of RCS and ROC.

Next, AMI patients were divided into the low SHR group (*n* = 208) and high SHR group (*n* = 133). The incidence of MACCEs and death during hospitalization increased significantly in the high SHR group, when compared to the low SHR group (Table 2).

**Table 2:** In-hospital outcomes in low and high SHR groups.

**In-hospital outcomes**	**Low SHR**	**High SHR**
	** <1.25 (*n* = 208)**	**≥1.25 (*n* = 133)**
MACCEs	27 (12.98%)	42 (31.58%)
All-cause death	17 (8.17%)	27 (20.30%)
Cardiogenic shock	19 (9.13%)	34 (25.56%)
Reinfarction	1 (0.48%)	2 (1.50%)
Mechanical complications of MI	5 (2.40%)	8 (6.01%)
Ventricular free wall rupture	4	3
Papillary muscle rupture	1	2
Ventricular septal rupture	0	3
Ischemic stroke	2 (0.96%)	4 (3.01%)
Major bleeding	5 (2.40%)	12 (9.02%)
Gastrointestinal bleeding	1	8
Cardiac tamponade	3	1
Retroperitoneal bleeding	1	1
Urinary bleeding	0	1
Access site bleeding	0	1

*MACCEs, major adverse cardiovascular and cerebrovascular events; MI, myocardial infarction; SHR, stress hyperglycemia ratio*.

### Logistic Analysis

**Figure 3:**
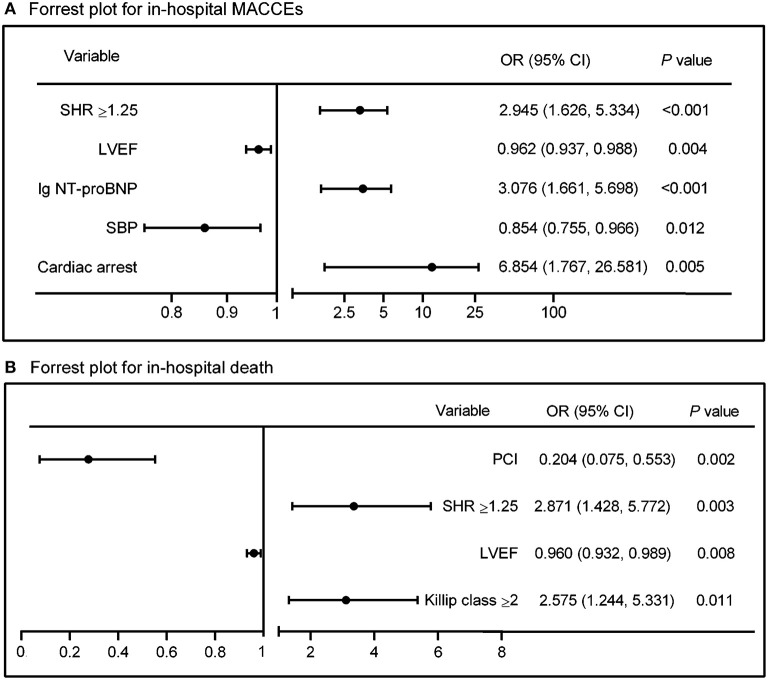
Forest plots showing risk factors for in-hospital **(A)** MACCEs and **(B)** mortality by multivariate logistic analysis. Summary estimates in **(A)** for SBP indicate OR per 10 mmHg increment in SBP. SHR, stress hyperglycemia ratio; LVEF, left ventricular ejection fraction; NT-proBNP, N-terminal pro-B type natriuretic peptide; SBP, systolic blood pressure; PCI, percutaneous coronary intervention; OR, odds ratio; CI, confidence incidence.

In univariate logistic analysis, cardiac arrest before admission, Killip class, heart rate, SBP, S2B, PCI or not, complete revascularization, NT-proBNP, LVEF, admission glucose as well as a SHR ≥ 1.25 significantly predicted in-hospital MACCEs (OR: 3.094, 95% CI: 1.794–5.337, *P* < 0.001, Table 3). After adjusting for the potential confounding factors in multivariate logistic analysis, including age, gender and above potential confounders, a high SHR remained independently associated with in-hospital MACCEs (OR: 2.945, 95% CI: 1.626–5.334, *P* < 0.001) (Table 3, Figure 3). In addition, a high SHR was associated with increased risk of in-hospital mortality (OR = 2.862, 95% CI= 1.492–5.491, *P* = 0.002); and the association remained significant after adjusting for potential confounding factors (OR: 2.871, 95% CI: 1.428–5.772, *P* = 0.003) (Table 4, Figure 3).

**Table 3:** Univariate and multivariate logistic regression analysis for in-hospital MACCEs.

**Variable**	**Univariate analysis**		**Multivariate analysis**^**a**^	
	**Odd ratios (95% CI)**	***P*-Value**	**Odd ratios (95% CI)**	***P*-Value**
Age	1.037 (0.974–1.105)	0.253		
Gender	0.621 (0.364–1.059)	0.080		
Hypertension	1.280 (0.712–2.299)	0.409		
Diabetes	1.618 (0.928–2.819)	0.090		
Cardiac arrest before admission	3.605 (1.171–11.100)	0.025	6.854 (1.767–26.581)	0.005
Killip class ≥ 2	2.632 (1.522–4.550)	0.001		
Heart rate	1.026 (1.011–1.042)	0.001		
Systolic blood pressure (per 10 mmHg)	0.829 (0.738–0.931)	0.002	0.854 (0.755–0.966)	
S2B within 12 h	0.530 (0.308–0.911)	0.022		0.012
Multi-vessel disease	0.933 (0.520–1.673)	0.815		
PCI	0.296 (0.124–0.708)	0.006		
Complete revascularization	0.488 (0.286–0.834)	0.009		
Hemoglobin	0.988 (0.975–1.001)	0.069		
Serum creatinine	1.002 (1.000–1.005)	0.080		
Lg NT-proBNP	3.944 (2.253–6.904)	<0.001	3.076 (1.661–5.698)	<0.001
LVEF	0.952 (0.930–0.974)	<0.001	0.962 (0.937–0.988)	0.004
Admission glucose (per mmol/L)	1.088 (1.023–1.157)	0.007		
SHR ≥ 1.25	3.094 (1.794–5.337)	<0.001	2.945 (1.626–5.334)	<0.001

a *Forward stepwise regression method was used in the multivariate logistic analysis, adjusted for age, gender, diabetes, cardiac arrest, Killip class, heart rate, systolic blood pressure, S2B, PCI, complete revascularization, LVEF, NT-proBNP, and admission glucose*.

*S2B, symptom onset to balloon time; PCI, percutaneous coronary intervention; NT-proBNP, N-terminal pro-B type natriuretic peptide; LVEF, left ventricular ejection fraction; SHR, stress hyperglycemia ratio; CI, confidence incidence*.

**Table 4:** Univariate and multivariate logistic regression analysis for in-hospital death.

**Variable**	**Univariate analysis**		**Multivariate analysis**^**a**^	
	**Odd ratios (95% CI)**	***P*-Value**	**Odd ratios (95% CI)**	***P*-Value**
Age	1.039 (0.964–1.119)	0.315		
Gender	1.645 (0.870–3.112)	0.126		
Hypertension	1.455 (0.705–3.002)	0.310		
Diabetes	1.290 (0.659–2.527)	0.458		
Cardiac arrest before admission	3.200 (0.942–10.875)	0.062		
Killip class ≥ 2	3.421 (1.720–6.804)	<0.001	2.575 (1.244–5.331)	0.011
Heart rate	1.029 (1.011–1.047)	0.001		
Systolic blood pressure (per 10 mmHg)	0.845 (0.736–0.969)	0.016		
S2B within 12 h	0.468 (0.241–0.908)	0.025		
Multi-vessel disease	0.804 (0.406–1.592)	0.531		
PCI	0.192 (0.078–0.477)	<0.001	0.204 (0.075–0.553)	0.002
Complete revascularization	0.485 (0.256–0.918)	0.026		
Hemoglobin	0.985 (0.970–1.000)	0.052		
Serum creatinine	1.002 (0.999–1.005)	0.169		
Lg NT-proBNP	3.542 (1.841–6.814)	<0.001		
LVEF	0.949 (0.923–0.976)	<0.001	0.960 (0.932–0.989)	0.008
Admission glucose (per mmol/L)	1.073 (1.001–1.150)	0.048		
SHR ≥ 1.25	2.862 (1.492–5.491)	0.002	2.871 (1.428–5.772)	0.003

a *Forward stepwise regression method was used in the multivariate logistic analysis, adjusting for age, gender, diabetes, Killip class, heart rate, systolic blood pressure, S2B, PCI, complete revascularization, LVEF, NT-proBNP, and admission glucose*.

*S2B, symptom-onset-to-balloon time; PCI, percutaneous coronary intervention; NT-proBNP, N-terminal pro-B type natriuretic peptide; LVEF, left ventricular ejection fraction; SHR, stress hyperglycemia ratio; CI, confidence incidence*.

When used as a continuous variable, SHR remained an independent predictor of in-hospital MACCEs (OR: 2.363, 95% CI: 1.127–4.954, *P* = 0.023) and in-hospital all-cause death (OR: 2.513, 95% CI: 1.153–5.476, *P* = 0.020) in multivariate analysis. In contrast, admission glucose concentrations were only significantly associated with adverse clinical outcomes in univariate analysis, but not after adjustments for above confounders (Supplementary Tables 1, 2).

In order to assess whether the association between SHR and in-hospital outcomes was robust if only patients underwent revascularization were included, we conducted a sensitivity analysis. The results showed that a high SHR value remained significantly associated with the risk of in-hospital adverse events (Supplementary Table 3).

### Subgroup Analysis: SHR in Diabetic and Non-diabetic Patients

Since the stress hyperglycemia may be influenced by long-term metabolic state, subgroup analysis was carried out between patients with and without diabetes. In non-diabetic patients, a SHR value over 1.25 was significantly associated with in-hospital MACCE and mortality, even after adjusting for potential confounding factors, such as age, gender, heart rate, Killip class, LVEF and NT-proBNP (Table 5). Nevertheless, SHR could not predict in-hospital outcomes (MACCEs or death) in diabetic patients in logistic analysis (Table 5).

**Table 5:** Subgroup analysis based on the diabetic and non-diabetic population.

**Subgroup**	**SHR <1.25**	**SHR ≥ 1.25**	**Univariate analysis**			**Multivariate analysis**^**a**^		
	**(No. of events/patients)**		**Odds ratio (95% CI)**	*P-*value^*^	***P* for interaction**	**Odds ratio (95% CI)**	*P*-value^*^	***P* for interaction^**†**^**
MACCEs								
Non-diabetic	19/162	24/79	3.704 (1.873–7.324)	<0.001	0.252	2.716 (1.281–5.762)	0.009^b^	0.358
Diabetic	9/46	17/54	1.889 (0.747–4.776)	0.179				
Deaths								
Non-diabetic	13/162	16/79	2.911 (1.323–6.407)	0.008	0.914	2.394 (1.040–5.507)	0.040^c^	0.930
Diabetic	4/46	11/54	2.686 (0.792–9.106)	0.113				

a *Forward stepwise regression method was used in the multivariate logistic analysis*.

b *Adjusted for age, gender, cardiac arrest, Killip class, heart rate, systolic blood pressure, S2B, PCI, complete revascularization, LVEF, and NT-proBNP*.

c *Adjusted for age, gender, Killip class, heart rate, systolic blood pressure, S2B, PCI, complete revascularization, LVEF, and NT-proBNP*.

* *P-value for SHR groups*;

† *P-value for interaction of groups with subgroups*.

*S2B, symptom onset to balloon time; PCI, percutaneous coronary intervention; NT-proBNP, N-terminal pro-B type natriuretic peptide; LVEF, left ventricular ejection fraction; SHR, stress hyperglycemia ratio; CI, confidence incidence*.

## Discussion

In the present study, we found that SHR, an index of SIH intensity, was independently associated with in-hospital MACCEs, and mortality in elderly patients with AMI. SHR levels correlated with in-hospital outcomes with a non-linear dose–response relationship. Subgroup analysis showed the outcome-predicting value of SHR was significant in patients without pre-existing diabetes, but not significant in those with diabetes. To our knowledge, this is the first report to examine the predictive significance of SHR in an elderly population with AMI.

Stress hyperglycemia is a common finding and a strong predictor for adverse clinical outcomes after AMI (4–6). The development of SIH may be attributed to a complex interplay of acute physiological changes, including increased gluconeogenesis, deleterious adrenergic activation, insulin resistance, and excessive counter-regulatory hormones, such as catecholamine, cortisol, and cytokines (20, 21). While in turn, SIH contributes to a vicious cycle by inducing an increase in inflammatory cytokines, oxidative stress, endothelial dysfunction, thrombosis, and ischemia-reperfusion injury, all of which could cause further cardiac damage (22–25). As a result of these complex reactions, a high admission SHR may reflect the severe alterations in the inflammatory, and hemodynamic status in AMI patients, especially in those complicated with serious complications such as cardiogenic shock or infection. Moreover, acute fluctuations in glucose levels are associated with increased plaque instability, infarct size, and worse heart function (26), which may also lead to worse prognosis. Also, recent studies reported that stress hyperglycemia was positively associated with in intracoronary thrombus burden and no-reflow phenomenon, which may further explain the significantly higher incidence of mortality and cardiogenic shock in high SHR group (27, 28).

Elderly patients are under-represented in previous studies and the predictive value of SIH in AMI has been quite inconsistent. In the Cooperative Cardiovascular Project (CCP) which enrolled 141,680 AMI patients older than 65 years, glucose at admission was associated with a steep linear mortality increase in non-diabetic patients (7). Another observational study by Nicolau et al. (8) indicated that admission glucose concentrations independently predicted in-hospital mortality in AMI patients, but the predictive value of SIH was less significant or even insignificant in patients older than 70 years. In our study consisted of a very old population, admission glucose was associated with adverse outcomes only in univariate analysis, but not after adjustments for potential confounders. These discrepancies may be attributed to the fact that admission glucose level does not always accurately reflect SIH intensity. In addition, elderly patients have higher incidence of complications, such as unrecognized diabetes, impaired β-cell function, malnutrition, and frailty (29), which may lead to changes in chronic glycemic status as well as impaired glucose response after injury (30). The main finding of this study is that SHR, an index that more accurately reflects the extent of SIH by correction for chronic glycemic status, independently predicted in-hospital outcomes. More importantly, the predictive value of SHR remained significant even after adjustments for confounding factors such as baseline health status, medical history, organ functions, or revascularization time, suggesting SHR may serve as a strong prognostic marker in the risk stratification for very old patients with AMI.

Our findings were in line with previous studies. Yang et al. (31) recently reported that SHR was a useful predictor of 30-day MACCEs (all-cause death, non-fatal MI and stroke) after PCI, especially in non-diabetic patients with AMI. Another observational study consisted of STEMI patients who were discharged alive revealed a significant correlation between high SHR and worse long-term prognosis in non-diabetic population, but the relationship was not significant in diabetic patients (18). Consistently, we found the outcome-predicting value of SHR was different between patients with and without diabetes in subgroup analysis. One possible explanation is that diabetes itself contributes to poor clinical outcomes, which may partly mask the effects of high SHR in this subgroup. On the other hand, the acute inflammatory and glycemic responses were more prominent in patients with newly diagnosed hyperglycemia than those with diabetes (32), since the correction speed of hyperglycemia in diabetic patients might have been readjusted over the chronic time (33). Nevertheless, the number of diabetic patients in our study was quite limited, thus whether SHR is of good predictive significance in elderly patients with DM needs to be further investigated in large-population studies with long follow-up.

Despite the strong association between hyperglycemia and AMI prognosis, the optimal treatments for stress hyperglycemia remains an unsettled question, especially in the elderly. Clinical trials of glucose lowering therapies with specific glucose targets yielded conflicting results. For example, the DIGAMI study demonstrated that intensive insulin therapy reduced all-cause mortality in AMI patients with stress hyperglycemia irrespective of the previous diabetes status (34). Conversely, a meta-analysis of 3 trials revealed limited benefits of intensive glucose control in AMI patients with diabetes, but a significantly increased risk of serious hypoglycemia (35). In addition, elderly patients always exhibit the poorest glycemic control but the highest risk of hypoglycemia during acute phase of AMI (36), making it difficult to define glucose-controlling targets in this subgroup. As SHR was shown to be a stronger predictor of poor prognosis, we propose that stratified glycemic targets based on SHR values rather than the absolute glucose value may be applied to the management of SIH in future studies.

There are several limitations in our study. Firstly, we cannot exclude the possibility of selection bias, because subjects lacking stress-induced glucose or HbA1c values were excluded. Secondly, the sample size is relatively limited, which may partly mask the predictive significance of SHR in diabetic patients. Thirdly, we only evaluated the relationship between SHR and in-hospital outcomes, and a long-term follow-up will provide a more comprehensive assessment of SHR. Albeit, our study is still of critical clinical importance, since life-threatening complications often occurs during the acute phase of MI especially in the elderly and SIH had a more significant relationship with the short-term than long-term prognosis in previous studies (31, 37). Our data need to be interpreted with caution, and further studies with a large population, longer follow-up and prospective evaluation are needed to confirm the role of SHR in very old patients with AMI.

## Conclusion

SHR, an index to reflect intensity of stress hyperglycemia, is a simple and strong predictor of in-hospital outcomes in elderly patients with AMI, especially in non-diabetic population. Prospective studies are warranted to investigate whether glycemic control using SHR as a target could improve clinical outcomes in elderly patients with or without DM.

## Data Availability Statement

The raw data supporting the conclusions of this article will be made available by the authors, without undue reservation.

## Ethics Statement

The studies involving human participants were reviewed and approved by the Ethics Committee of Guangdong Provincial People's Hospital. Written informed consent for participation was not required for this study in accordance with the national legislation and the institutional requirements.

## Author Contributions

GC and ML wrote the manuscript and conducted statistical analysis. XW and RW conducted data inspection and validation. YZ provided funding support and supervision. LX and XH designed the study and revised the manuscript. All authors contributed to the article and approved the submitted version.

## Conflict of Interest

The authors declare that the research was conducted in the absence of any commercial or financial relationships that could be construed as a potential conflict of interest.
