# Supplementary material for: Association Between Stress Hyperglycemia Ratio and In-hospital Outcomes in Elderly Patients With Acute Myocardial Infarction
Source: Front Cardiovasc Med. 2021 Jul 20;8:698725. doi: 10.3389/fcvm.2021.698725 (PMC8329087; doi:10.3389/fcvm.2021.698725)
Supplement: Supplementary file 1 [file Data_Sheet_1.pdf]

Supplementary Table 1. Univariate and multivariate logistic regression analysis for in-hospital MACCEs

Variable	Univariate analysis		Multivariate analysis[a]	
	Odd ratios (95% CI)	*P*-Value	Odd ratios (95% CI)	*P*-Value
Age	1.037 (0.974–1.105)	0.253		
Gender	0.621 (0.364–1.059)	0.080		
Hypertension	1.280 (0.712–2.299)	0.409		
Diabetes	1.618 (0.928–2.819)	0.090	2.233 (1.158–4.307)	0.016
Cardiac arrest before admission	3.605 (1.171–11.100)	0.025	6.655 (1.755–25.243)	0.005
Killip class ≥ 2	2.632 (1.522–4.550)	0.001		
Heart rate	1.026 (1.011–1.042)	0.001	1.019 (1.002–1.036)	0.031
Systolic blood pressure (per 10mmHg)	0.829 (0.738–0.931)	0.002	0.834 (0.737–0.944)	0.004
S2B within 12h	0.537 (0.313–0.924)	0.025		
Multi-vessel disease	0.933 (0.520–1.673)	0.815		
PCI	0.296 (0.124–0.708)	0.006		
Complete revascularization	0.488 (0.286–0.834)	0.009		
Hemoglobin	0.988 (0.975–1.001)	0.069		
Serum creatinine	1.002 (1.000–1.005)	0.080		
Lg NT-proBNP	3.944 (2.253–6.904)	<0.001	2.960 (1.589–5.512)	0.001
LVEF	0.952 (0.930–0.974)	<0.001	0.967 (0.941–0.993)	0.013
Admission glucose (per mmol/L)	1.088 (1.023–1.157)	0.007		
SHR[b]	3.267 (1.716–6.223)	<0.001	2.363 (1.127–4.954)	0.023

Notes: [a] Forward stepwise regression method was used in the multivariate logistic analysis, adjusting for age, gender, diabetes, cardiac arrest, Killip class, heart rate, systolic blood pressure, S2B, PCI, complete revascularization, LVEF, NT-proBNP, and admission glucose.

Abbreviations: S2B, symptom onset to balloon time; PCI, percutaneous coronary intervention; NT-proBNP, N-terminal pro-B type natriuretic peptide; LVEF, left ventricular ejection fraction; SHR, stress hyperglycemia ratio; CI, confidence incidence.

Supplementary Table 2. Univariate and multivariate logistic regression analysis for in-hospital death

Variable	Univariate analysis		Multivariate analysis[a]	
	Odd ratios (95% CI)	*P*-Value	Odd ratios (95% CI)	*P*-Value
Age	1.039 (0.964–1.119)	0.315		
Gender	1.645 (0.870–3.112)	0.126		
Hypertension	1.455 (0.705–3.002)	0.310		
Diabetes	1.290 (0.659–2.527)	0.458		
Cardiac arrest before admission	3.200 (0.942–10.875)	0.062		
Killip class ≥ 2	3.421 (1.720–6.804)	<0.001	2.757 (1.337–5.687)	0.006
Heart rate	1.029 (1.011–1.047)	0.001		
Systolic blood pressure (per 10mmHg)	0.845 (0.736–0.969)	0.016		
S2B within 12h	0.468 (0.241–0.908)	0.025		
Multi-vessel disease	0.804 (0.406–1.592)	0.531		
PCI	0.192 (0.078–0.477)	<0.001	0.231 (0.088–0.606)	0.003
Complete revascularization	0.485 (0.256–0.918)	0.026		
Hemoglobin	0.985 (0.970–1.000)	0.052		
Serum creatinine	1.002 (0.999–1.005)	0.169		
Lg NT-proBNP	3.542 (1.841–6.814)	<0.001		
LVEF	0.949 (0.923–0.976)	<0.001	0.963 (0.935–0.991)	0.011
Admission glucose (per mmol/L)	1.073 (1.001–1.150)	0.048		
SHR[b]	2.869 (1.374–5.989)	0.005	2.513 (1.153–5.476)	0.020

Notes: [a] Forward stepwise regression method was used in the multivariate logistic analysis, adjusting for age, gender, diabetes, Killip class, heart rate, systolic blood pressure, S2B, PCI, complete revascularization, LVEF, NT-proBNP, and admission glucose.

Abbreviations: S2B, symptom-onset-to-balloon time; PCI, percutaneous coronary intervention; NT-proBNP, N-terminal pro-B type natriuretic peptide; LVEF, left ventricular ejection fraction; SHR, stress hyperglycemia ratio; CI, confidence incidence.

Supplementary Table 3. Association between SHR and in-hospital outcomes in sensitivity analyses (Excluding patients without coronary revascularization, n = 318)

	Univariate analysis		Multivariate analysis[a]	
	Odds ratio (95% CI)	*P*- value	Odds ratio (95% CI)	*P*-value
MACCEs				
SHR <1.25	1 (reference)			
SHR ≥ 1.25	2.990 (1.670–5.354)	< 0.001	2.568 (1.384–4.767)	0.003[b]
Death				
SHR <1.25	1 (reference)			
SHR ≥ 1.25	2.957 (1.429–6.119)	0.003	2.374 (1.107–5.090)	0.026[b]

Notes: [a]Multivariate logistic analysis with forward stepwise regression method. [b]Adjusted for age, gender, diabetes, cardiac arrest, Killip class, heart rate, systolic blood pressure, S2B, complete revascularization, LVEF, NT-proBNP, and admission glucose.
